# Editorial: Global excellence in ethnopharmacology: Africa

**DOI:** 10.3389/fphar.2025.1616493

**Published:** 2025-06-11

**Authors:** Hellen Oketch-Rabah, David R. Katerere

**Affiliations:** ^1^ United States Pharmacopeial Convention, Rockville, MD, United States; ^2^ Tshwane University of Technology, Pretoria, South Africa

**Keywords:** African traditional medicine (ATM), ethnopharmacology, botanical, herbal, sub-Saharan Africa, healthcare

African Traditional Medicine (ATM) has a rich history of use in the continent dating back to the dawn of humanity. This knowledge has been preserved and passed down through generations via oral traditions and today ATM continues to thrive, with traditional healers outnumbering conventional doctors and pharmacists in sub-Saharan Africa. Thus, traditional medicine remains a vital, though often overlooked, component of the continent’s healthcare system.

At the heart of ATM are the plants that form the foundation of traditional healing practices. Many of these plants have deep roots in African ethnobotany. For example, the aloe family, particularly *Aloe vera* (L.) Burm. f. (syn *Aloe barbadensis* Mill.) (originally misattributed to the West Indies) and *Aloe ferox* Mill. (Cape aloes), have played a significant role in both traditional and global medicine. *A. ferox* was once the only species with an official monograph in the British Pharmacopoeia, while gum arabica obtained from *Senegalia senegal* L. Britton (syn *Acaia senegal* (L.) Willd.) from East Africa remains a key ingredient in pharmaceuticals and consumer goods. Africa’s plant diversity comprises approximately 45,000 vascular plant species, including 6,300 endemic to South Africa’s Cape region and 8,600 to Madagascar.

Despite this rich botanical and cultural heritage, Africa’s contribution to global pharmaceutical medicine remains limited. Some important drugs like tubocurarine, camptothecin, and yohimbine were derived from African medicinal plants. However, much of the contemporary research in ethnopharmacology by African scientists remain under published, often confined to academic theses publications available only in hard copy at universities. Efforts to publish these findings in international journals or commercialize them into herbal or pharmaceutical products are fragmented, mainly hampered by a lack of research infrastructure and funding.

This Research Topic on Global Excellence in Ethnopharmacology in Africa has highlighted groundbreaking studies on the pharmacological and biological effects of plants, fungi, animals, microorganisms, and minerals used in ATM for both human and livestock health. The Research Topic features nine high-quality articles from across the continent, including five original research papers and four review articles (Mambou et al.; Beressa et al.; Nyazema et al.; Okumu et al.; Setlhare et al.; Brendler et al.; Pretorius and Smith, 2023; Irungu et al.; Smith et al. Topics covered range from the anti-epileptic effects of *Mimosa pudica* L. extracts to ethnobotanical studies in Ethiopia and Kenya, immunological research on South Africa’s anti-HIV product Nkabinde, and the well-known herbal remedy Umckaloabo from Lesotho. The review papers focus on disease conditions critical to Africa including malaria, COVID-19, as well as on translational ethnomedicine, and the use and misuse of psychoactive plants.

These contributions offer a glimpse into the vibrant and multidisciplinary field of ethnopharmacology in Africa. They lay the groundwork for future research, particularly in areas that would further the development of these botanicals for purposes such as commercial exploitation, intellectual property rights, access, and benefit-sharing, and improving access to medicines for African patients. This is especially crucial in an era of diminishing resources for global health, where traditional medicine can play a pivotal role in addressing healthcare challenges and needs.

Ethnopharmacological research in Africa should focus not only on documenting the known herbal materials and practises but also on developing herbal based products to improve the health of its people. Presently the depth of such research is questionable as there is often use of inappropriate *in vitro* models and minimal progression in the use of newer techniques of compound isolation and new approach methodologies (NAMs) for the assessment of efficacy and safety of these natural products. Research on herbal products is acutely hampered by the lack of good laboratory infrastructure and strategic collaborations. In countries like South Africa, stringent regulations governing the research on indigenous genetic resources have pushed researchers and potential collaborators to refocus their research efforts on non-indigenous species or botanicals from other countries that maybe more welcoming of such research.

Thus, in the context of all this what is required is capacity building for the next-generation of researchers. This could be achieved by working with some of the existing organizations interested in natural product research such as the Association of African Medicinal Plant Standards (AAMPS), GA – African Research Network, NAPRECA, in association with journals such as Frontiers. Capacity building should focus on the key pillars of pharmacognosy, i.e., taxonomy and documentation of African flora and ethnobotanical use, phytochemistry, pharmacological and toxicological research (including the use of appropriate and valid assays) and commercial development and regulatory science of herbal products.

